# Hydrogel-based reprogramming of the tumor inflammatory microenvironment: from mechanotransduction and dynamic materials to adaptive immunotherapy

**DOI:** 10.3389/fimmu.2026.1947516

**Published:** 2026-10-06

**Authors:** Jiansong He, Juan Wei, Jiemei Chen, Heng Long, Taotao Liu, Weipeng Wei

**Affiliations:** 1 Department of Pharmacy, The First Affiliated Hospital of Guangxi Medical University, Nanning, China; 2 Department of Traditional Chinese Medicine, Qinzhou Maternal and Child Health Care Hospital, Qinzhou, China

**Keywords:** drug delivery, dynamic covalent chemistry, hydrogel, inflammation, mechanotransduction, tumor microenvironment

## Abstract

Modulating inflammation within the tumor microenvironment (TME) has emerged as a crucial avenue in contemporary cancer therapy. However, the intricate signaling networks, mechanical heterogeneity, spatiotemporal drug gradients, and poor immune cell infiltration continue to hinder the effectiveness of conventional therapeutic strategies. Hydrogels, with tunable physicochemical properties and versatile biological functions, offer a promising platform for addressing these challenges. This review first unpacks the inflammatory pathways active in the TME and then provides a structured overview of hydrogel classifications and material characteristics. From there, it discusses how mechanotransduction pathways (e.g., YAP/TAZ, Piezo1, FAK) and chemical signaling frameworks shape the interaction between hydrogels and the TME. In addition, the article highlights hydrogels’ translational potential by outlining their roles in inflammation-associated biomarker detection, precision drug delivery systems, and *in vivo* compatibility evaluations. Recent advances in dynamic covalent hydrogels, DNA-based hydrogels, living hydrogels, and stimuli-responsive systems are critically reviewed to clarify the field’s technological direction. Their integration with immunotherapy is particularly promising but raises concerns about long-term biosafety, degradation product toxicity, and immune compatibility. Despite their considerable promise for precisely modulating TME inflammation, hydrogels face persistent challenges that must be addressed to enable clinical potential. Overcoming these hurdles will require sustained multidisciplinary efforts. We further propose a stage-aware immunomodulatory framework, in which programmable hydrogels can coordinate mechanical, metabolic, and immune regulation according to the evolving biological state of the TME. Here, stage-aware does not imply a universal temporal hierarchy of TME remodeling, but rather refers to adaptive coordination of multiple regulatory modules according to tumor-specific biological states. This concept moves beyond single-pathway modulation and aligns with the emerging paradigm of “adaptive immunotherapy” that dynamically responds to TME evolution.

## Introduction

The tumor microenvironment (TME) functions as a highly dynamic ecosystem, consisting not only of malignant tumor cells but also of stromal and immune cell populations interwoven with a complex extracellular matrix (ECM) (1, 2). Among the non-malignant components, cancer-associated fibroblasts (CAFs) and a variety of immune cells form an interactive cellular network that can either facilitate or restrict tumor progression, depending on contextual cues (3). Emerging evidence suggests that metabolic regulation is central to this process, serving as a key medium through which cells communicate and exert reciprocal influence, ultimately shaping both tumor growth and anti-tumor immune responses (4). Recent findings further highlight that mechanical forces transmitted via the ECM can directly modulate immune cell function–a concept termed “mechanomedicine.” For instance, matrix stiffness regulates YAP/TAZ nuclear translocation in macrophages and T cells, thereby influencing their polarization state and cytotoxic activity. Hydrogels, by mimicking tissue-specific stiffness, offer a powerful platform to study and exploit these mechano-immune interactions (5, 6).

Such communication occurs through the direct exchange of metabolites, as well as through non-metabolic signaling routes—including cytokine release, ECM remodeling, and hypoxia-driven pathways—which together drive metabolic reprogramming in surrounding cell populations (7). To illustrate, immune cells such as T cells, B cells, tumor-associated macrophages (TAMs), and neutrophils engage in intricate crosstalk with tumor cells, thereby influencing processes like lymphatic dissemination (8). In parallel, structural and supportive constituents of the TME, including endothelial cells, adipocytes, and ECM components, contribute to tumor progression by secreting pro-angiogenic factors that remodel the local niche in favor of malignancy (8, 9). From this perspective, the TME should be regarded not as a passive scaffold but as a metabolically active and constantly adapting micro-ecosystem that dictates the trajectory of tumor evolution.

Within this intricate microenvironment, inflammation functions as a pivotal regulatory force that shapes tumor initiation, progression, and even the ways in which cancer cells escape immune surveillance (10). Rather than being a mere byproduct of malignancy, chronic inflammation actively contributes to nearly every stage of tumorigenesis—from the earliest cellular transformation to later metastatic dissemination (11, 12). In the initial phase of tumor formation, inflammatory signaling can foster the emergence and expansion of precancerous clones by inducing DNA lesions and, importantly, by impairing the corresponding repair pathways. A typical example can be observed in inflammation-driven malignancies such as inflammatory bowel disease–related colorectal cancer, where sustained inflammatory stress markedly elevates cancer susceptibility (13). As the disease advances, inflammation supports tumor growth through multiple converging mechanisms. One major route involves the stimulation of angiogenesis, which secures a continuous supply of oxygen and nutrients to proliferating tumor cells (14). Cytokines released by infiltrating inflammatory cells can activate endothelial cell proliferation and migration, thereby fueling the formation of new vasculature (15). At the same time, inflammatory mediators can tilt the immune balance toward suppression, enabling tumor cells to effectively bypass immune clearance. Cells residing within the TME secrete immunosuppressive molecules such as interleukin-10 (IL-10), dampening the cytotoxic responses of T cells and natural killer cells (16). Furthermore, inflammatory cues can trigger epithelial–mesenchymal transition (EMT), granting tumor cells enhanced motility and invasive capacity, which ultimately accelerates metastatic spread (17). Beyond classical cytokines, emerging research implicates the NLRP3 inflammasome and gasdermin D (GSDMD)-mediated pyroptosis, as well as ACSL4-dependent ferroptosis, as key drivers of inflammation-associated tumor progression. These pathways represent new targets for hydrogel-based intervention (18, 19).

Hydrogels, as a highly promising class of biomaterials, possess numerous significant advantages. They exhibit excellent biocompatibility, enabling harmonious coexistence with biological tissues and reducing immune and inflammatory responses, which gives them extensive application prospects in the field of biomedicine (20, 21). Hydrogels also demonstrate adjustable mechanical properties; by altering their composition and preparation conditions, their mechanical characteristics can be tailored to resemble various human soft tissues, ranging from soft tissues such as skin to relatively stiff cartilage (22–24). Furthermore, hydrogels are stimuli-responsive, reacting to temperature, pH, light, and electric or magnetic fields, thereby enabling controlled drug release or alterations in material properties (25). For instance, certain polysaccharide-based magnetic hydrogels can achieve targeted drug delivery under the influence of external magnetic fields, enhancing therapeutic efficacy (26). Recent advances in TME-responsive biomaterials further indicate that material chemistry itself can actively participate in microenvironmental regulation. In particular, metal–phenolic coordination systems and polyphenol-based hydrogels combine stimulus-responsive behavior with modular therapeutic functions, providing additional strategies for modulating hypoxic, inflammatory, and immunosuppressive tumor microenvironments (27, 28).

This review establishes a material-to-immunity framework to understand how hydrogel properties regulate inflammatory and immune responses within the TME. Within this framework, we use the term “stage-aware” to describe adaptive coordination of multiple regulatory modules according to tumor-specific biological states rather than a predetermined temporal hierarchy of TME remodeling. Rather than focusing solely on hydrogel classification or therapeutic cargo, we discuss how physicochemical characteristics, including chemical composition, mechanical properties, network architecture, and degradation behavior, function as biological regulators that influence inflammatory signaling, metabolic remodeling, and immune-cell states. We further examine how these material-derived signals are translated into therapeutic strategies, including immune modulation, combination immunotherapy, and adaptive hydrogel systems. Finally, we critically discuss current limitations and future opportunities toward personalized and clinically translatable hydrogel-based cancer therapies.

To orient the reader, the overall conceptual framework of this review is schematically illustrated in **Figure 1**. The figure depicts how locally administered hydrogels form an in situ matrix within the TME, interact with tumor cells, immune cells, CAFs, and ECM components, and thereby reshape inflammatory and immunosuppressive signaling. The resulting therapeutic effects include tumor-cell apoptosis and necrosis, ECM degradation or remodeling, macrophage repolarization from an M2-like to an M1-like phenotype, dendritic-cell maturation, and enhanced immune-cell infiltration. In parallel, hydrogel-mediated treatment may promote the release of DAMPs such as HMGB1 and ATP, regulate immunosuppressive mediators including IL-10 and TGF-β, and enhance antitumor immunity through IFN-γ signaling and PD-1/PD-L1 checkpoint blockade. **Figure 1** thus provides a visual roadmap for the material-to-immunity framework discussed throughout this review.

**Figure 1:**
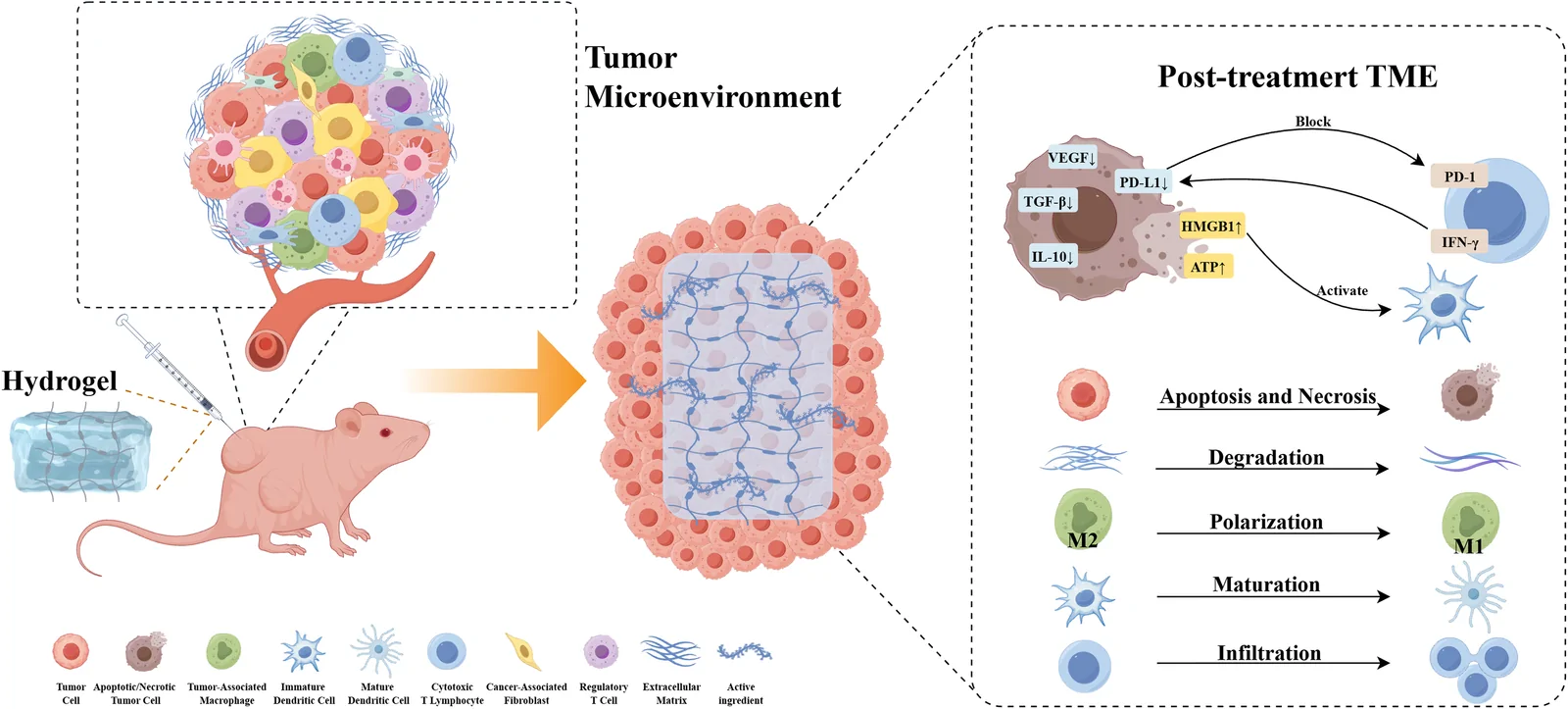
Hydrogel-mediated modulation of the TME and post-treatment effects. This figure illustrates the role of hydrogel injection in modulating TME *in vivo*. Visualizations were created using FigDraw. The left panel depicts the initial TME, including tumor cells, TAMs, dendritic cells, cytotoxic T lymphocytes, cancer-associated fibroblasts, regulatory T cells, and the extracellular matrix. The hydrogel is administered by injection and forms a local matrix within the tumor, where it interacts with cellular and structural components of the TME, as illustrated in the central panel. The right panel summarizes representative post-treatment effects, including tumor-cell apoptosis and necrosis, extracellular-matrix degradation or remodeling, macrophage polarization from an M2-like to an M1-like phenotype, dendritic-cell maturation, and enhanced immune-cell infiltration. Hydrogel-mediated treatment may also promote the release of damage-associated molecular patterns (DAMPs), such as HMGB1 and ATP, regulate immunosuppressive mediators including IL-10 and TGF-β, and enhance antitumor immune responses through pathways such as IFN-γ signaling and PD-1/PD-L1 checkpoint blockade. Overall, the schematic highlights the potential of hydrogels to regulate inflammatory and immune responses within the TME, thereby supporting the development of cancer immunotherapy strategies.

Recent preclinical studies have increasingly focused on combining hydrogels with immunotherapeutic regimens, such as immune checkpoint inhibitors (ICIs), chimeric antigen receptor (CAR) T cells, and STING agonists (29, 30). These combinations leverage the hydrogel’s ability to localize immune modulators within the tumor bed, thereby amplifying anti-tumor immunity while dramatically reducing systemic immune-related adverse events (irAEs) (29). Although still in early stages, this integrated approach has shown durable responses in multiple syngeneic and xenograft models, positioning hydrogels as a promising adjunct to next-generation cancer immunotherapy (31).

The literature search for this review was conducted using the PubMed and Web of Science databases. In addition, preprint repositories (bioRxiv, medRxiv) were screened to capture recent unpublished advances (2023–2025). A combination of keywords-including ‘hydrogels’, ‘tumor microenvironment (TME)’, ‘inflammation’, ‘cancer therapy’ and ‘immunomodulation’ -and their synonyms was used. Boolean operators (AND/OR) were applied to refine the queries. The search was restricted to publications from 2012 to the present. Inclusion criteria were as follows: original research articles and reviews published in English that specifically addressed hydrogel-based strategies for modulating inflammation within the TME. Studies were excluded if they focused on non-inflammatory aspects of the TME or did not involve direct application of hydrogels. Title/abstract screening, followed by full-text review and data extraction, was conducted to ensure the relevance of the studies to hydrogel design, mechanisms, and therapeutic outcomes.

## Characteristics of inflammation in the TME and its regulatory significance

### Biological basis of tumor-associated inflammation

Tumor-associated inflammation has a complex biological foundation. Tumor cells interact with immune cells, stromal cells, and other components within the TME, forming an inflammatory milieu. TAMs are key regulatory cells in this inflammatory environment, with their phenotype and function influenced by TME (32, 33). For example, cytokines secreted by tumor cells, such as colony-stimulating factor-1 (CSF-1), can recruit monocytes and promote their differentiation into TAMs, which often exhibit M2-like polarization, thereby promoting tumor growth, immune suppression, and angiogenesis (34, 35). Recent studies have uncovered that lactate accumulation in the TME drives histone lactylation, a novel epigenetic modification that promotes M2-like gene expression and immunosuppression. Hydrogel-delivered lactate oxidase can disrupt this process, offering a metabolic reprogramming strategy (36, 37).

Beyond classical cytokines, emerging research implicates the NLRP3 inflammasome and GSDMD-mediated pyroptosis, as well as ACSL4-dependent ferroptosis, as key drivers of inflammation-associated tumor progression (38). NLRP3 activation in TAMs promotes IL-1β and IL-18 secretion, fostering an immunosuppressive and pro-angiogenic niche. Hydrogels loaded with MCC950 (a selective NLRP3 inhibitor) or with ferroptosis inducers (e.g., sulfasalazine, iron oxide nanoparticles) can exploit these pathways to convert inflammatory cues into anti-tumor signals (39). Such strategies remain largely unexplored and represent a future direction for hydrogel-based TME reprogramming.

Additionally, CAFs play an important role in tumor-associated inflammation (40, 41). CAFs secrete various cytokines and chemokines that regulate the recruitment and function of inflammatory cells, thus promoting tumor occurrence and development (42). For instance, in certain tumors, IL-6 secreted by CAFs can activate the signal transducer and activator of transcription 3 (STAT3) pathway, promoting the proliferation and survival of tumor cells (43).

Importantly, CAFs are heterogeneous: inflammatory CAFs (iCAFs) produce abundant IL-6 and CXCL1, whereas myofibroblastic CAFs (myCAFs) generate dense matrix. These subsets exhibit distinct responses to hydrogel stiffness and degradation cues, which should be considered in material design (44). Meanwhile, ECM components in the TME, such as hyaluronic acid (HA), are closely linked to tumor-associated inflammation, particularly under metabolic dysregulation (45). Degradation products of HA can activate inflammatory signaling pathways, further exacerbating inflammation (46).

### The relationship between inflammation and tumor progression

The interplay between inflammation and tumor progression is both intimate and multifaceted. Persistent inflammatory activity shapes a microenvironment that actively supports tumor initiation and subsequent development by fostering tumor cell proliferation, enhancing survival signaling, and facilitating angiogenesis as well as metastatic spread (11, 47). In cancers driven or exacerbated by inflammation, the continuous release of reactive oxygen species (ROS) and pro-inflammatory cytokines by immune and stromal cells contributes to cumulative DNA damage and an increased mutational load, ultimately encouraging malignant transformation of susceptible cells (48). From this perspective, chronic inflammation should be regarded not merely as a bystander but as a driving force that gradually remodels local tissue biology in favor of oncogenic evolution.

Inflammation can also affect tumor progression by regulating immune cells in the TME. On one hand, inflammation can induce the aggregation and activation of immunosuppressive cells, such as regulatory T cells (Tregs) and myeloid-derived suppressor cells (MDSCs), which suppress anti-tumor immune responses and promote the immune evasion of tumor cells (48, 49). On the other hand, inflammation can activate anti-tumor immune cells, such as cytotoxic T lymphocytes (CTLs) and natural killer cells (NK cells); however, within the TME, this anti-tumor immune response is often suppressed. For instance, in some tumors, high concentrations of lactate in the inflammatory microenvironment can inhibit CTLs’ activity, preventing them from effectively killing tumor cells. Furthermore, inflammation can promote epithelial-mesenchymal transition (EMT) in tumor cells, enhancing their migration and invasion capacities, thereby facilitating tumor metastasis (17, 50). Matrix stiffening, a hallmark of many solid tumors, activates YAP/TAZ signaling, which synergizes with inflammatory cytokines to induce EMT (51). Hydrogels with tunable stiffness may modulate this process by providing mechanically defined niches (52).

### Therapeutic strategies for targeting inflammation regulation

Targeting inflammation regulation is an important strategy in cancer treatment. Small molecule inhibitors that target inflammation-related signaling pathways are among the commonly used methods. For example, inhibitors of the nuclear factor kappa-light-chain-enhancer of activated B cells (NF-κB) signaling pathway, such as [5-(p-fluorophenyl)-2-ureido] thiophene-3-carboxamide (TPCA-1), can suppress the expression of inflammatory factors, thereby alleviating tumor-associated inflammation (53). By loading TPCA-1 onto nanoparticles and functionalizing them, specific binding to endothelial cells and macrophages at the inflammation sites can be achieved, enabling targeted therapy for inflammation.

Furthermore, modulating immune cell function is also a crucial strategy for targeted inflammation regulation. For instance, adjusting macrophage polarization from the tumor-promoting M2 type to the anti-tumor M1 type can enhance the anti-tumor immune response. Additionally, monoclonal antibodies targeting inflammation-related cytokines also show potential in cancer therapy. For example, adalimumab, a monoclonal antibody against tumor necrosis factor-α (TNF-α), blocks TNF-α signaling and attenuates inflammatory responses, achieving notable efficacy in the treatment of certain inflammation-associated tumors (54).

However, macrophage polarization within tumors is not a binary M1/M2 process. TAM states exist along a continuous spectrum influenced by cytokine gradients, metabolic signals, hypoxia, and mechanical cues. Therefore, hydrogel-mediated macrophage regulation should be evaluated based on functional changes, such as antigen presentation capacity, inflammatory mediator production, and interaction with other immune populations, rather than relying solely on M1/M2 classification.

## Fundamental properties and classification of hydrogels

### Definition of hydrogels

Hydrogels are three-dimensional (3D) polymer networks composed of hydrophilic chains cross-linked physically or chemically that absorb and retain large amounts of water without dissolving (55). Hydrogels are widely used in tissue engineering, drug delivery, and TME studies due to their high water content, compliance, and physicochemical similarity to the native ECM (55–57). Beyond serving as structural scaffolds that support cell adhesion and growth, hydrogels actively modulate inflammatory mediators, immune-cell behavior, and signaling pathways through their chemical composition, physical properties, and degradation profiles, thereby providing distinctive value for regulating inflammation within the TME and for augmenting immunotherapy (58).

### Sources and classification

Hydrogels are commonly classified by origin as natural, synthetic, or hybrid/composite (59). Natural hydrogels—such as hyaluronic acid, alginate, gelatin, chitosan, and collagen—exhibit excellent biocompatibility and degradability and partially retain the bioactivity of their native counterparts. For example, hyaluronic acid binds CD44 receptors to regulate cell migration and inflammatory responses (60), whereas chitosan possesses intrinsic antimicrobial and immunomodulatory properties attributable to its amino groups (61). Although widely used to build tissue-mimetic platforms and delivery systems, natural hydrogels often display insufficient mechanical strength and batch-to-batch variability (62). Synthetic hydrogels, exemplified by poly(ethylene glycol) (PEG) (63) and polyacrylamide (64), offer fine control of stiffness, degradation rate, and drug-release kinetics through molecular-weight selection, cross-link-density design, and surface modification. These materials show high reproducibility and structural stability and are therefore attractive for clinical potential, though their degradation products can raise biosafety concerns (65). Hybrid/composite hydrogels integrate the advantages of natural and synthetic matrices and often incorporate functional components—such as inorganic nanoparticles, metal ions, or photosensitive small molecules—to enhance mechanics or impart stimulus responsiveness (66). For instance, graphene oxide–reinforced hydrogels (67) exhibit increased mechanical strength and enable photothermal therapy. Despite their functional integration, hybrid systems typically require complex fabrication, and scale-up is frequently limited by batch inconsistency and constrained process control.

### Hydrogel network formation and stimulus-responsive remodeling

Hydrogel network formation and subsequent remodeling are two related but distinct processes. Network formation determines the initial structural integrity and mechanical properties of hydrogels, whereas stimulus-responsive remodeling regulates how established networks adapt to tumor microenvironmental cues.

### Physical cross-linking

Physically cross-linked networks rely on noncovalent interactions (hydrogen bonding, hydrophobic association, ionic interactions, and van der Waals forces), enabling rapid gelation under mild conditions and often reversibility (68, 69). For instance, alginate forms stable gels in the presence of Ca^2+^. However, such networks typically display limited mechanical robustness and stability, constraining their suitability for long-term implantation.

### Chemical cross-linking

In contrast, chemical cross-linking creates covalent bonds between polymer chains through approaches such as free-radical polymerization, click reactions, and enzymatic cross-linking (68). These hydrogels exhibit greater strength and stability; pore size and degradation kinetics can be tuned via cross-link density, enabling long-term use and controlled release. Nevertheless, synthesis may leave residual cross-linkers or by-products.

### Dynamic covalent cross-linking and adaptive remodeling

A particularly exciting recent development is dynamic covalent hydrogels, which utilize reversible covalent bonds (e.g., boronate esters, imines, and disulfides) to enable self-healing, shear-thinning behavior, and adaptive network remodeling. These materials can dynamically reorganize their network structures in response to physiological or pathological cues within the TME, thereby supporting prolonged and context-responsive drug delivery (74, 75).

Unlike static covalent networks, dynamic covalent hydrogels (DCHs) contain reversible covalent linkages (e.g., boronate esters, imines, disulfides, and acylhydrazones) that undergo exchange reactions under physiological conditions, allowing the network properties to adapt over time (76). This dynamic bond exchange endows DCHs with unique properties, including self-healing, shear-thinning behavior, stress relaxation, and tunable network remodeling kinetics (77). In the context of TME inflammation, DCHs can integrate reversible bond exchange with endogenous TME cues (such as pH, ROS, and ATP fluctuations) to regulate network properties and drug release behavior. For example, dynamic covalent systems have been explored for ROS-responsive or context-sensitive delivery of immunotherapeutic agents (78). Recent studies have demonstrated that DCHs incorporating dual dynamic bonds (boronic ester + imine) can achieve reversible gel-sol transitions and tunable network adaptation, making them promising platforms for sustained and context-sensitive immunomodulation (79).

### Stimulus-responsive network remodeling

After network formation, stimulus-responsive hydrogels can undergo programmed remodeling, degradation, or cargo release in response to TME signals or external stimuli (70)—for example, acid-triggered dissolution of chitosan networks (71), reactive-oxygen-species-mediated disulfide cleavage (72), or near-infrared irradiation that breaks photosensitive groups (73). Such ‘smart’ hydrogels support precise drug delivery and spatiotemporally controlled therapy.

### Key physicochemical properties and signaling regulation functions

Hydrogel properties are governed not only by composition and crosslinking but also by key physicochemical attributes.

Chemical composition and functional groups (80, 81) modulate interactions with cells and the microenvironment; for example, carboxyl, amino, and sulfate groups can bind receptors or proteins and trigger inflammatory or immune signaling.

Surface charge (82) shapes interactions with cell membranes and extracellular macromolecules: cationic hydrogels associate readily with negatively charged membranes, enhancing cellular uptake. In addition, ligand presentation, such as RGD peptides, antibody fragments, or other specific ligands, enables targeted and cell type selective engagement.

Mechanical properties (83, 84), notably stiffness and viscoelasticity, strongly regulate cellular behavior: lower stiffness can bias macrophages toward an M1 like state and heighten immune activity, whereas higher stiffness favors M2 like polarization and angiogenesis; greater viscoelasticity more closely mimics native tissues and helps stabilize the local microenvironment.

Network architecture (85), including pore size, mesh size, and permeability, governs diffusion of drugs and signaling factors as well as immune cell migration; macroporous structures facilitate cell infiltration and mass exchange, whereas dense, small pore networks better support sustained release.

Degradation kinetics also influence hydrogel performance by controlling structural stability and therapeutic duration (86). Beyond degradation rate, degradation-derived products may also exert biological effects within the TME. Therefore, hydrogel degradation should be carefully designed to balance material stability, therapeutic function, and biological safety (87).

The preceding sections describe the fundamental physicochemical properties of hydrogels—composition, crosslinking type, mechanical behavior, and degradation kinetics—that define their potential for TME modulation. A central question remains: how do these static material properties translate into functional regulation of inflammatory signaling in the TME?

In the following section, we address this question from both passive and active perspectives. From the passive perspective, we examine how hydrogel physicochemical properties serve as a “signal-docking interface” that engages inflammatory mediators, chemokines, and immune cell receptors through chemical groups, charge distribution, and pore architecture. From the active perspective, we explore how mechanical signals—stiffness and viscoelasticity—are transduced through Piezo1, FAK, and YAP/TAZ pathways to actively reprogram immune cell function. This dual perspective shifts the discussion from static material description to dynamic material–biology interaction, and provides the mechanistic basis for the therapeutic applications discussed later.

### Interaction mechanisms between hydrogels and the TME

#### Physicochemical properties - inflammatory signal docking model

Inflammatory signaling within the TME is highly complex. Owing to their physicochemical properties, hydrogels can act not only as carriers but also as interfaces for signal regulation. On this basis, we propose an inflammatory signal docking model to describe dynamic interactions between hydrogel matrices and signaling molecules. Through their chemical groups, charge distribution, pore architecture, and degradability, hydrogels engage inflammatory mediators, chemokines, and immune cell receptors, thereby reshaping signal transduction networks (88). For example, negatively charged hyaluronic acid hydrogels modulate CXCL12/CXCR4 signaling via the HA/CD44 axis (89, 90), and sulfated polysaccharide hydrogels mimic the cytokine reservoir function of the ECM by binding heparin-binding domains to store and release cytokines and chemokines (91, 92). Thus, hydrogels constitute an inflammatory signal docking platform that can both passively carry and release signaling molecules and actively modulate signaling pathways through their intrinsic structural features.

#### Mechanical properties and immune regulation

The mechanical properties of hydrogels—particularly stiffness and viscoelasticity—are key modulators of immune status in the TME. Lower stiffness has been associated with M1-like inflammatory features and elevated IL-12/TNF-α in some systems, while higher stiffness correlates with M2-like immunosuppressive phenotypes in certain models (93). However, such associations are highly context-dependent, influenced by tumor type, ECM composition, ligand presentation, cytokine milieu, and cellular origin; they should not be interpreted as universal stiffness rules, nor can they be reduced to a simple M1/M2 binary. Viscoelasticity further affects immune-cell infiltration and retention—greater viscoelasticity can enhance T-cell penetration and sustain effector function (94). Therefore, mechanical properties should be viewed as modulatory signals rather than deterministic polarizing cues, and hydrogel performance should be assessed by functional immune outcomes (cytokine production, antigen presentation, immune-cell recruitment, and tumor control) rather than by polarization markers alone.

At the molecular level, matrix mechanics engage multiple mechanosensitive pathways: stiffness modulates T-cell activation via Piezo1 and YAP/TAZ, while macrophages sense mechanical cues through integrin-FAK and ROCK signaling. Physiological-like mechanical environments often correlate with enhanced immune activation, whereas excessive stiffening may promote immunosuppression and angiogenesis—again, these outcomes depend on the specific material context and should not be generalized as fixed stiffness thresholds. These mechanosensitive routes are not isolated; they form an integrated mechanochemical network (95, 96). Hydrogel-derived mechanical signals are first sensed by membrane-associated mechanosensors (integrin-FAK, Piezo1), which regulate cytoskeletal tension and downstream YAP/TAZ activity, subsequently influencing inflammatory mediator production, immune-cell activation, and metabolic adaptation. Conversely, inflammatory remodeling and stromal activation can reciprocally alter ECM mechanics, creating a feedback loop that couples hydrogel properties, mechanotransduction, and immune regulation—a complexity that underscores the need for context-aware material design.

#### Chemical composition and degradation products

Unlike the previously discussed physicochemical properties that regulate hydrogel–cell interactions, degradation-derived products represent a distinct biological regulatory layer after hydrogel breakdown.

These degradation products can influence inflammatory responses within the TME. Hyaluronic acid fragments can modulate inflammatory responses through Toll-like receptor 4 (TLR4) signaling (97). Degradation products can also participate in metabolic processes within the TME: oligosaccharides generated during hyaluronic acid degradation promote angiogenesis and inflammation (98), whereas acidic metabolites released from polymers based on lactate exacerbate local acidosis and enhance the metabolic advantage of tumor cells (99). By incorporating responsive degradation mechanisms—such as pH responsive or ROS cleavable bonds—hydrogels can coordinate drug release with metabolic regulation (100, 101).

More recently, “active degradation” has emerged as a design principle: hydrogels containing disulfide bonds release glutathione-scavenging byproducts that induce ferroptosis in tumor cells, while iron-chelating hydrogels can simultaneously inhibit ferroptosis and reduce inflammation. Thus, degradation should be viewed not merely as a fate but as a therapeutic opportunity (18, 19). For instance, disulfide-containing hydrogels have been reported to deplete GSH and induce ferroptosis in cancer cells (19). Conversely, iron-chelating hydrogels (e.g., deferoxamine-modified alginate) can inhibit ferroptosis and reduce inflammation in chronic wounds (102). These contrasting examples highlight that hydrogel degradation may influence biological outcomes rather than merely represent a terminal material fate. Beyond degradation-specific design, broader polymer-engineering strategies, including PEGylation, may further optimize drug delivery performance and therapeutic efficacy (103).

The preceding sections describe the mechanisms by which hydrogels interact with the TME across four dimensions: (i) physicochemical signal docking—chemical and charge-based engagement of inflammatory mediators; (ii) mechanotransduction—stiffness and viscoelasticity sensed via Piezo1, FAK, and YAP/TAZ; (iii) compositional regulation—functional group and ligand presentation modulating cell behavior; and (iv) degradation-mediated signaling—bioactive breakdown products participating in inflammatory cascades. Collectively, these analyses converge on one conclusion: hydrogels act not as passive carriers but as active interfaces that participate in signal transduction and immune regulation.

The next section builds on this mechanistic foundation to examine four therapeutic strategies enabled by these principles: anti-inflammatory factor delivery, pro-inflammatory microenvironment remodeling, immune checkpoint modulation, and immune adjuvant delivery. This section thus transitions from mechanistic understanding to therapeutic design, and demonstrates how each application strategy derives from the interaction mechanisms described above.

### Application strategies of hydrogels in regulating inflammation in the TME

Targeting inflammatory dysregulation and immunosuppression in the TME is essential for improving the response rate and durability of cancer therapy (11). Clinical studies indicate that polarization of TAMs toward the M2 phenotype, accumulation of Tregs, and elevated levels of immunosuppressive cytokines such as IL-10 and TGF-β are associated with poor responses to immunotherapy and to radiotherapy and chemotherapy (104). Consequently, hydrogel platforms that accumulate at lesion sites, release payloads in a cue- and time-controlled manner, and interact with pathological signals have emerged as promising tools to remodel inflammatory and immune states in the TME.

In particular, responsive delivery systems tuned to TME features (low pH, high glutathione, specific proteases, ROS, or hypoxia) enable site-specific dosing while reducing systemic exposure and toxicity (105, 106). Moreover, as understanding of the inflammation–immunity–metabolism interplay deepens, hydrogels have progressed from passive carriers to active regulators that integrate signal amplification, immune reprogramming, and combination therapy (107).

Early work primarily loaded chemotherapeutic agents into hydrogels to increase local drug concentration and limit systemic toxicity (108). For example, an injectable self-healing chitosan/poly(vinyl alcohol) (CS/PVA) hydrogel achieved pH-dependent retention and release of 5-fluorouracil (5-FU) (108): leakage into normal tissues was minimized near neutral pH, whereas sustained release occurred under acidic conditions, thereby enhancing antitumor efficacy and reducing adverse effects. As research advanced, the immunoregulatory value of hydrogels became evident (109): by loading immunomodulatory factors, oligonucleotides, or small molecules with programmable release, hydrogels can upregulate proinflammatory and antitumor pathways, suppress immunosuppressive axes, and reconstruct antitumor immune circuits. A representative example is an injectable lipid-nanoparticle hydrogel composite (LPR@CHG) which, in a pancreatic cancer model, increased IRF5 and decreased CCL5, driving TAM polarization toward M1 and inhibiting tumor growth (110). Moreover, hydrogel-based three-dimensional tumor models enable analysis of dynamic interactions among cells, the ECM, and inflammatory signals, and can be used to screen candidate therapeutic regimens (111).

### Anti-inflammatory factor delivery systems based on hydrogels

To correct the imbalance between proinflammatory and immunosuppressive cues in the TME, hydrogels can establish favorable cytokine gradients at lesion sites by delivering anti-inflammatory or immunoregulatory agents locally, efficiently, and in a sustained manner. For example, a GelMA–PEG composite hydrogel with integrated microfluidic channels enables repeated infusion of IL-4 or dexamethasone (DEX) after implantation and provides sustained release through the channels, lowering TNF-α and IL-6 expression and shifting macrophage phenotype; within an appropriate dose window, this platform balances inflammation resolution with tissue repair (112). Triggered, on-demand release is likewise important: chitosan-based hydrogels loaded with rhein sustain drug release under acidic conditions characteristic of the TME, inhibit MAPK signaling (JNK, ERK, and p38), reduce TNF-α and IL-1β, and thereby attenuate the proinflammatory cascade, while localized delivery limits systemic adverse effects (113).

Recent advances have introduced mRNA-loaded hydrogels that transiently express anti-inflammatory cytokines (IL-10, IL-1RA) directly at the tumor site, avoiding systemic cytokine toxicity. Similarly, exosome-loaded hydrogels (e.g., MSC-derived exosomes) have shown potent macrophage reprogramming and T cell activation in preclinical models, providing a cell-free immunomodulatory platform (114, 115).

### Pro-inflammatory microenvironment remodeling strategies

Reprogramming entails not only dampening inflammation but, more importantly, reversing immunosuppression and restoring antitumor immunity. In oncology research, interpenetrating polymer network (IPN) hydrogels dynamically regulate local cytokine gradients through ion release and matrix remodeling, thereby creating conditions that support immune cell activation (116). Likewise, pH responsive sodium alginate (SA) hydrogel nanoemulsions release immunomodulatory molecules in acidic microenvironments, reducing TNF-α and IL-6 while increasing IL-10. Although first validated in inflammatory models, this mechanism—acid triggered release coupled with cytokine balancing—closely reflects the microenvironment of inflammation-associated tumors such as colorectal cancer and informs immune reprogramming strategies (117). In solid tumor models, immunomodulatory hydrogels delivering IL-12 or STAT3 inhibitors promote polarization of TAMs toward the M1 phenotype, enhance antitumor immunity, and attenuate the immunosuppressive activity of M2 TAMs.

A newer strategy involves delivering lactate oxidase or pyruvate kinase M2 (PKM2) inhibitors via hydrogels to reduce lactate accumulation, thereby relieving T cell suppression and restoring NK cell activity (118). These metabolic interventions synergize with immune checkpoint blockade.

### Hydrogel-mediated immune checkpoint modulation

Immune checkpoint blockade (ICB) is limited by local immunosuppression and systemic toxicity, whereas hydrogels that combine localized, sustained release with lesion-site accumulation can enhance intra-tumoral efficacy while reducing required doses (29). Thermosensitive or photosensitive networks can encapsulate agents such as anti–PD-1, anti–PD-L1, or anti–CTLA-4 antibodies and release them in response to body temperature or external stimuli, maintaining effective concentrations at the lesion, increasing tumor-infiltrating lymphocyte (TIL) infiltration and activity, and reducing immune-related adverse events. A more advanced triple-modality approach—integrating radiotherapy or chemotherapy, ICB, and hydrogels—uses therapy-induced ATP release and immunogenic cell death (ICD) as triggers; concurrently, the hydrogels deliver immune adjuvants (e.g., CpG) to amplify innate immunity and antigen cross-presentation, producing synergistic tumor clearance and durable memory responses in established solid-tumor models (119).

Beyond membrane-associated PD-L1, tumor-derived exosomal PD-L1 represents an additional mechanism of local immunosuppression and resistance to PD-1/PD-L1 blockade. A metal–phenolic network incorporating the exosome inhibitor GW4869 and Fe³^+^-mediated ferroptosis demonstrated that suppression of exosomal PD-L1 could restore T-cell activity, while photothermal therapy-associated immunogenic cell death promoted dendritic-cell maturation and further enhanced antitumor immunity (120). Although this platform is not a hydrogel, it highlights a transferable strategy for future hydrogel-based immunotherapy in which extracellular-vesicle-mediated checkpoint signaling and tumor-cell death pathways are simultaneously regulated.

In addition, bispecific T-cell engager (BiTE)-loaded hydrogels have achieved localized retargeting of T cells to tumor cells while minimizing systemic cytokine release syndrome (121). Furthermore, CRISPR-based hydrogel systems enabling localized immune-cell engineering, including PD-1 editing strategies, represent an emerging approach for improving checkpoint immunotherapy (122).

### Hydrogels as delivery platforms for immune adjuvants

Implantation of immune adjuvants such as CpG, Toll-like receptor (TLR) agonists, or stimulator of interferon genes (STING) agonists into immunologic niches is an effective strategy for converting immune-cold tumors into immune-inflamed tumors. For example, an in situ–gelling alginate system that incorporates an ATP aptamer and CpG can form an intra-tumoral reservoir: ATP released by radiotherapy or chemotherapy triggers CpG dissociation and uptake by dendritic cells, thereby enhancing antigen presentation and T-cell priming (121). Certain polysaccharide or short peptide hydrogels also display intrinsic immunomodulatory activity. Chitin-based thermosensitive gels used as vaccine adjuvants increase cytokines associated with Th1 and Th2 responses (123), whereas injectable gels designed with L-norvaline as a functional component suppress the ARG1 pathway and mitigate immunosuppression (124).

More recently, STING agonist-loaded hydrogels (e.g., cGAMP, MSA-2) have shown remarkable ability to convert “cold” tumors into “hot” tumors by activating the cGAS-STING pathway, which upregulates type I interferons and promotes dendritic cell maturation (125, 126). Combining STING agonists with hydrogel delivery may reduce systemic exposure while sustaining intratumoral STING activation (127, 128). Beyond synthetic adjuvants, exosome-loaded hydrogels have emerged as a cell-free immunomodulatory platform. Mesenchymal stem cell (MSC)-derived exosomes possess intrinsic anti-inflammatory and tissue-regenerative properties (129). When loaded into injectable hydrogels (e.g., hyaluronic acid or gelatin methacryloyl), exosomes can be released in a sustained manner, reprogramming TAMs toward an M1-like phenotype and enhancing CD8+ T cell infiltration (130). Compared to direct exosome injection, hydrogel encapsulation improves local retention, reduces clearance by the reticuloendothelial system, and avoids off-target accumulation (131). Beyond hydrogel-based delivery, preclinical models of melanoma and colon cancer have shown that neoantigen-painted exosomes synergize with anti-PD-1 therapy, achieving complete remission in a subset of animals (132).

### Multifunctional responsive hydrogels

Programmable hydrogels that respond to multiple, synergistic stimuli (temperature, pH, light, magnetic or electric fields, enzymes, and ROS) provide a versatile platform for precise regulation of the TME. Among these systems, intelligent polyphenol-based hydrogels represent an emerging class of TME-responsive materials. Their characteristic crosslinking chemistry can confer responsiveness to endogenous cues such as pH, reactive oxygen species, and glutathione, while also providing intrinsic adhesive, antioxidant, anti-inflammatory, and immunomodulatory properties. These features make polyphenol-based hydrogels particularly attractive for localized drug release and dynamic remodeling of the immunosuppressive TME (28). The carboxymethyl cellulose–N-isopropylacrylamide–acrylamide (CMC–NIPAM–AM) interpenetrating network exhibits dual responsiveness, releasing cargo at physiological temperature and tumor-associated pH (133). A polysaccharide–PCA/DFO system that employs dual dynamic bonds (boronic ester and imine) integrates adhesion, self-healing, and glucose responsiveness and shows antioxidant, antibacterial, and immunomodulatory activity (134). In addition, cold atmospheric plasma (CAP) induces *in situ* gelation of HA–tyramine loaded with nano-adjuvants that block TGF-β (TLN@CHG), which enhances dendritic-cell activation and infiltration of CD4+ and CD8+ T cells, resulting in substantial clearance of the primary tumor and protection upon tumor rechallenge in a colon cancer model (135).

### Living hydrogels: opportunities, biosafety challenges, and translational perspectives

An emerging frontier is “living hydrogels”, which incorporate engineered microorganisms (e.g., E. coli Nissle 1917) within hydrogel matrices to provide dynamic biological functions and adaptive regulation of the TME (136). These bacteria can be programmed to secrete immunomodulatory proteins (IL-2, GM-CSF) or therapeutic molecules involved in inflammatory regulation (e.g., arginase inhibitors) in response to TME signals, potentially enabling adaptive and sustained therapeutic activity (137). The hydrogel matrix can provide a supportive niche that may improve local retention of engineered microorganisms; however, it should not be considered a complete biocontainment strategy. Potential concerns include microbial escape, uncontrolled proliferation, genetic instability, and unpredictable interactions with host immunity. Future development of living hydrogels will therefore require robust containment strategies, including genetic safeguards, programmable control circuits, and long-term monitoring of host–microbe interactions.

These approaches illustrate the convergence of biomaterials, synthetic biology, and immunotherapy, opening new avenues for next-generation cancer treatments. Nevertheless, compared with conventional acellular hydrogels, living hydrogel systems face additional regulatory and manufacturing challenges, including reproducible production, quality control, sterilization compatibility, and evaluation of long-term safety. Addressing these issues will be essential for clinical translation.

### Tumor-targeted delivery technologies based on hydrogels

A combined strategy that integrates targeting ligands, triggered prodrugs, and *in situ* gelation offers an efficient route to enhance lesion accumulation and tissue penetration. CRGDK-modified doxorubicin prodrug nanoparticles (PDNPs) undergo thermally induced *in situ* self-assembly to form a degradable hydrogel depot (PDNPs-gel), leveraging neuropilin-1 (NRP-1) targeting and tumor acidity to achieve deep penetration and intracellular release; this system markedly suppresses recurrence in models of incomplete breast-cancer resection (138). A hyaluronic acid–mesoporous silica–gold (HA-MSNs-Au) nanocomposite platform enables photoinduced gelation, using HA–CD44 affinity for tumor-selective adhesion and *in situ* self-assembly under near-infrared (NIR) irradiation; subsequently, upregulated hyaluronidase (HAase) drives a closed-loop sequence of enzymatic degradation, drug release, and cellular reuptake (117).

### Novel hydrogel platforms and fabrication technologies

Several hydrogel platforms exemplify programmed, cascade immunomodulation in the TME. TED-Gel is designed to deliver payloads in sequence: initial doxorubicin release induces immunogenic cell death (ICD) and converts dead tumor cells into autologous vaccines; subsequent CpG delivery activates antigen-presenting cells; material degradation and payload composition promote macrophage reprogramming from M2-like to M1 phenotypes; and delayed anti–PD-L1 release helps restore exhausted T cell function. In preclinical studies, this sequence suppressed primary, distant, and rechallenge tumors (139). In postoperative models, COS@LA hydrogels combine antitumor, anti-inflammatory, and antibacterial functions; sustained release modulated AKT phosphorylation and reduced TNF-α and IL-6, prolonging tumor-free survival and lowering recurrence risk (140). Recent additive manufacturing (AM) strategies—including extrusion- and inkjet-based 3D printing—enable spatially patterned hydrogel architectures with tunable porosity and on-demand multi-agent loading, thereby strengthening structure–function control in TME-oriented designs (141–143).

These examples illustrate the feasibility of temporally programmed delivery, but such programming reflects sequential coordination of therapeutic modules rather than a validated universal hierarchy of mechanotransduction, metabolism, and immunity; thus, it should be viewed as a design strategy requiring further verification. Advanced fabrication techniques, including volumetric printing and two-photon polymerization, now enable construction of TME-mimetic organoids with sub-micrometer precision, yet their clinical translation is hindered by GMP-related obstacles—sterilization methods (gamma irradiation, ethylene oxide, supercritical CO_2_) can degrade bioactive components or alter mechanical properties, and batch consistency for multi-component hydrogels remains challenging. Future efforts must prioritize continuous manufacturing and inline quality control to overcome these barriers (144–146).

The application strategies discussed above—encompassing anti-inflammatory factor delivery, pro-inflammatory microenvironment remodeling, immune checkpoint modulation, and immune adjuvant delivery—collectively illustrate the remarkable versatility of hydrogels as platforms for TME inflammation modulation. These strategies have demonstrated substantial preclinical efficacy across a range of tumor models, validating the therapeutic potential of hydrogel-based immunomodulation.

However, the journey from laboratory proof-of-concept to clinical implementation involves far more than simple dose escalation. The success of any hydrogel-based strategy is profoundly influenced by the specific characteristics of the target tumor type—including anatomical accessibility, stromal density, immune landscape (immune-cold versus immune-inflamed), and the extent of hypoxia and fibrosis. A strategy that proves effective in a highly vascularized, immunologically “hot” tumor may fail entirely in a densely fibrotic, immune-excluded tumor such as pancreatic cancer.

Therefore, in the following section, we move beyond general principles to examine how hydrogel design must be tailored to the constraints and opportunities of individual tumor types. We systematically discuss the characteristic TME features of major solid tumors, the feasibility of different administration routes, and the key design considerations for each context, with an emphasis on progress toward clinical translation and remaining challenges. This tumor-type-specific analysis completes the full logical chain of this review: from fundamental material design principles → mechanistic interaction understanding → general therapeutic strategies → context-specific clinical application.

## Roles in different tumor types and clinical application prospects

Applications of hydrogels across solid tumor types share common features but are constrained by tumor-specific microenvironments. Key advantages include establishing high local drug concentrations with controlled release at the lesion to reduce systemic toxicity; interfacing with tumor-microenvironment cues (acidity, hypoxia, enzymes) to enable on-demand release; and synergizing with immunotherapies—such as ICB and adjuvants—to enhance T cell infiltration and function. Differences arise from the immune landscape (immune-cold versus immune-inflamed tumors), the extent of stromal fibrosis, the severity of hypoxia, and anatomical accessibility, all of which determine administration routes, material design (e.g., stiffness, pore size, degradation), and the attainable depth of immune reprogramming. Evidence from systematic reviews and recent studies supports these conclusions: local hydrogel delivery increases intra-tumoral drug accumulation and lowers toxicity, and is particularly suitable for combination with immunotherapy to mitigate immune-related adverse events (irAEs) associated with systemic administration.

To further illustrate how hydrogel design is tailored to the constraints and opportunities of individual tumor types, **Table 1** summarizes the characteristic TME features, administration feasibility, key design concepts, and representative advances for several solid tumors, along with their progress toward clinical potential.

**Table 1:** Hydrogel-mediated TME inflammation-regulating strategies and clinical translation progress across different tumor types.

Tumor type	Characteristic TME features	Hydrogel administration feasibility	Key design concepts and strategies	Representative advances/evidence
Colorectal cancer (CRC)	CRC lesions often exhibit low immune infiltration and a locally immunosuppressive microenvironment.	Feasibility is moderate. Intratumoral injection or other local administration routes can be used.	Hydrogels can combine local chemotherapy with immunomodulation to remodel the TME.	Hydrogel-based treatment has been shown to remodel the CRC TME and enhance antitumor efficacy (147).
Triple-negative breast cancer (TNBC, cold tumor tendency)	TNBC often exhibits marked immunosuppression, high metastatic potential, and a tendency toward an immune-cold phenotype.	Feasibility is moderate. Hydrogels can be administered intratumorally or into the postoperative cavity.	Local STING agonist delivery can be combined with cell therapy to achieve multicomponent immune synergy.	A translatable platform integrating local STING delivery with cell therapy has been reported (147).
Hepatocellular carcinoma (HCC)	HCC is characterized by severe immunosuppression and immune tolerance, and focal ablation may be followed by an immune-cold state.	Feasibility is moderate. Intraoperative or post-ablation intracavitary administration is possible.	Post-ablation hydrogels can locally deliver innate immune activators, such as STING agonists, to reverse TAM polarization.	A STING-agonist hydrogel applied to residual foci after RFA remodeled the TME and enhanced antigen presentation (148).
Glioblastoma (GBM)	GBM is characterized by the blood-brain barrier and a strongly immunosuppressive TME.	Feasibility is moderate. Postoperative intracavitary implantation provides a practical local administration route.	Postoperative hydrogels can provide sustained local delivery of ICB agents, immune adjuvants, or oxygen-releasing components while improving local retention.	Hydrogel-based strategies have been used to reprogram STING signaling in CNS tumors (149).
Pancreatic cancer (highly fibrotic)	Pancreatic cancer is characterized by a dense extracellular matrix, severe hypoxia, and poor tissue permeability.	Administration is challenging and generally requires intraoperative placement or precise puncture.	Long-acting hydrogels with low diffusion rates can be combined with oxygen-releasing or anti-fibrotic modules to improve penetration and immune accessibility.	Local composite hydrogels have been combined with chemotherapy and immunotherapy (30).
Urothelial tumors (UTUC/NMIBC)	These tumors have favorable luminal accessibility and a prominent inflammation-epithelium axis.	Feasibility is high. Antegrade or retrograde perfusion permits local hydrogel administration.	Thermoreversible hydrogels can prolong drug residence, improve local inflammatory control, and reduce recurrence.	UGN-101 (Jelmyto, mitomycin C hydrogel) has received FDA approval, whereas multiple Phase III trials of UGN-102 have demonstrated high complete-response rates and durability (112, 113).

## Advantages and limitations of hydrogels in TME research

Hydrogels have significant advantages in TME research. They can simulate various characteristics of the TME, providing an excellent platform for in-depth studies on the interactions between tumor cells and TME components (cells, ECM, signaling molecules). For example, by adjusting the composition and structure of hydrogels, one can precisely control their mechanical properties and biochemical signals to mimic the microenvironment of different types of tumors (111). Hydrogels can also serve as drug delivery carriers, enabling localized, sustained, and precise modulation of the TME. By loading anti-inflammatory factors and immunomodulators, hydrogels can continuously release drugs at the tumor site, modulating the inflammatory response and immune status (112). Beyond these fundamental advantages and limitations, hydrogel systems exhibit multifaceted capabilities and constraints across five critical dimensions, as systematically evaluated in **Table 2**.

**Table 2:** Evaluation dimensions, advantages, limitations, and recent solutions of hydrogels in the regulation of TME.

Evaluation dimension	Advantages	Limitations	Representative solutions/cases
Precision biomimetic capability	Hydrogel composition, crosslinking density, and functional modification can be tuned to reproduce key mechanical properties, biochemical cues, and hypoxic conditions of the TME. This tunability supports 3D organoid culture and TME interaction studies (150).	Current hydrogel models cannot fully reproduce dynamic ECM remodeling, spatial gradients of immune-cell infiltration, or the temporal and spatial heterogeneity of physicochemical cues such as pH and enzyme activity.	Modular hydrogels, such as MELH systems integrating GSH-, pH-, and enzyme-responsive units, can dynamically adapt to changing TME conditions (148).
Immune regulation function	CA IX-targeted hydrogels can restrict tumor-derived exosome diffusion, thereby limiting immune evasion and enhancing CD8+ T-cell infiltration (111).	Hydrogel chirality can influence immune responses. D-chiral peptide hydrogels (D-Gel) have been reported to induce T-cell exhaustion and local immunosuppression.	L-chiral peptide hydrogels (L-Gel) can be used to reduce immune-suppressive effects, while low-immunogenic polymers such as polyethylene glycol can further improve immune compatibility.
Metabolic intervention capability	Hydrogels can target metabolic vulnerabilities in tumor cells. For example, 3M Gel inhibits LAT transporters and S-adenosylmethionine synthesis, whereas MELH depletes glucose and glutathione to disrupt redox balance (148, 151).	Tumor cells may compensate by activating alternative metabolic pathways, such as serine synthesis. Broad metabolic perturbation may also adversely affect stromal-cell functions.	Multi-pathway strategies can reduce metabolic compensation. For example, 3M Gel simultaneously blocks methionine uptake and intracellular metabolism (148).
Exosome regulation	Nanofiber hydrogels such as PepABS-py can form on tumor-cell membranes and physically intercept exosome release, thereby reducing distant metastatic signaling (152).	In highly invasive tumors such as gliomas, exosome secretion may exceed the capture capacity of hydrogels. Prolonged interception may also trigger escape mutations.	Combining CA IX inhibition with exosome-intercepting strategies may alleviate hypoxia and reduce baseline exosome secretion (152).
Clinical translation potential	Hydrogels are adaptable to minimally invasive administration. For example, subcutaneous hydrogel vaccines based on GBM lysates have been explored for recurrence prevention (152).	Clinical translation is limited by the lack of scalable sterile manufacturing and standardized quality-control procedures. Efficacy also remains insufficiently validated in large-animal models because most studies are limited to mice.	Patient-derived preclinical screening platforms, including PDX models, can be used to validate hydrogel efficacy before clinical translation.

However, there are also limitations to hydrogels in TME research. The efficacy of hydrogel-based TME regulation is highly context dependent. Differences in tumor type, stromal composition, immune landscape, administration route, and experimental model can substantially influence therapeutic outcomes. For example, a hydrogel formulation that effectively enhances immune infiltration in an immune-inflamed tumor may show limited benefit in immune-excluded or highly fibrotic tumors. Furthermore, many studies rely on simplified *in vitro* systems or small-animal models, which may not fully reproduce the spatial heterogeneity, mechanical properties, and immune complexity of human tumors. Establishing standardized evaluation criteria and clinically relevant models will therefore be essential for accurately assessing hydrogel performance.

The safety and controllable biodegradability of hydrogel materials are critical factors that must be carefully considered in their application for TME inflammation modulation. In terms of safety, although many hydrogels possess good biocompatibility, some crosslinkers or additives used in the synthesis of certain hydrogels may pose potential risks of cytotoxicity or adverse immune reactions (153). Regarding biodegradability, an ideal hydrogel should degrade into non-toxic or low-toxicity metabolic products at a controllable rate after fulfilling its therapeutic function to avoid long-term retention that could lead to foreign body reactions, encapsulation, or interference with normal tissue function (154). However, the degradation rates and mechanisms of different hydrogels can vary greatly. A degradation rate that is too rapid may result in burst release of drugs, reducing therapeutic efficacy and increasing the risk of side effects, while a degradation rate that is too slow may lead to prolonged retention of materials in the body, raising the risk of potential complications. For instance, hydrogels based on natural polymers usually exhibit good biodegradability, but their degradation rates may be difficult to control precisely (62). Therefore, rational regulation of hydrogel composition and network structure remains essential for achieving application-specific degradation profiles.

### Long-term biosafety and degradation product toxicity

The effects of long-term implantation of hydrogels in the TME have not been fully elucidated and remain an important focus and controversy in current research. On one hand, hydrogels may have positive long-term effects on the TME, such as continuously regulating the inflammatory state and promoting immune responses, thereby inhibiting tumor growth and metastasis (109). Some studies have shown that certain hydrogels loaded with immunomodulators can maintain a localized immune activation state for an extended period, helping to control tumor recurrence and metastasis (155). On the other hand, long-term implanted hydrogels may pose potential risks. For instance, their degradation products or sustained physical/chemical stimuli may have unknown effects on tumor cells or stromal cells, theoretically increasing the possibility of promoting tumor growth or invasion (156), necessitating in-depth long-term safety assessments. Additionally, long-term implantation of hydrogels may trigger immune responses, leading to issues such as inflammation or fibrosis, which can affect treatment outcomes and patients’ quality of life.

Long-term implantation studies (≥6 months) in large animal models are still scarce (157). Recent evidence indicates that some degradation products—such as PEG oligomers—can accumulate in lysosomes and induce chronic inflammation, while high-molecular-weight HA fragments may paradoxically promote tumor recurrence via TLR4 signaling (158, 159). These findings underscore the need for systematic toxicological profiling of degradation byproducts, including their effects on the gut-liver axis and systemic immune homeostasis.

Additionally, sterilization methods significantly affect hydrogel properties: gamma irradiation can cause additional crosslinking or chain scission; ethylene oxide leaves toxic residues; and aseptic processing is costly and scale-limited (160). Supercritical CO_2_ sterilization has emerged as a promising low-temperature alternative, but its compatibility with diverse hydrogel chemistries requires further validation (161). From a Good Manufacturing Practice (GMP) perspective, critical quality attributes (CQAs)—including gelation time, swelling ratio, pore size distribution, residual crosslinker content, and sterility—must be defined, and real-time process analytical technology (PAT) should be implemented for batch release (162).

### Sterilization and GMP manufacturing

The efficacy and stability of hydrogels in inflammation regulation are key issues for their application. In terms of efficacy, although numerous preclinical studies have confirmed the potential of hydrogels to regulate inflammation in the TME, significant differences exist in the actual therapeutic effects of different hydrogel systems in the complex *in vivo* environment (163). For example, some hydrogels exhibit good inflammation modulation capabilities *in vitro*, but their efficacy in the complex physiological environment *in vivo* may be limited by suboptimal drug release kinetics, insufficient targeting efficiency, immune evasion due to tumor heterogeneity, and the status of the host immune system. Therefore, it is necessary to further optimize the design of hydrogels to enhance their efficacy *in vivo*, such as through precise control of drug release and increased targeting efficiency to improve inflammation regulation outcomes. In terms of stability, hydrogels must maintain their structural integrity and functional activity in the challenging TME (e.g., low pH, high enzyme concentration, hypoxia, mechanical stress) to ensure the continuity and reliability of treatment (164). However, the complexity of the TME, such as low pH and high enzyme concentrations, may lead to structural damage or loss of function of the hydrogels. For example, certain hydrogels may degrade or deform under the acidic conditions of the TME, affecting their drug loading capacity and drug release kinetics. Therefore, it is crucial to develop hydrogels with good stability that can withstand the effects of various factors in the TME, maintaining their structural and functional stability for effective inflammation regulation.

Sterilization remains a major hurdle for clinical potential. Gamma irradiation can crosslink or fragment hydrogel polymers, altering drug release kinetics (160); ethylene oxide may leave toxic residues; aseptic processing is costly and scale-limited (165). Supercritical CO_2_ sterilization has emerged as a promising low-temperature alternative, but its compatibility with various hydrogel chemistries requires further validation (166). From a GMP perspective, CQAs such as gelation time, swelling ratio, pore size distribution, and residual crosslinker content must be defined, with real-time PAT for batch release (167).

## Tumor heterogeneity and individualized response

The same hydrogel may provoke divergent responses in different tumor types or even patients due to variations in stromal density, mutational burden, and baseline immune infiltrate (74). For example, a hydrogel that efficiently polarizes TAMs in lung adenocarcinoma may fail in desmoplastic pancreatic cancer due to dense stroma limiting penetration (168). The use of patient-derived xenograft (PDX) models and tumor organoids integrated with hydrogels is now enabling personalized screening of hydrogel formulations (169), and artificial intelligence (AI) models are being trained to predict optimal hydrogel design parameters from patient omics data (170).

## Future perspectives of hydrogels in regulating inflammation within the TME

### Potential of hydrogels in personalized cancer treatment

Hydrogel-based platforms offer opportunities for personalized cancer therapy. By precisely designing the composition, structure, and properties of hydrogels, specific responses and treatments can be achieved for TME of different patients. For example, hydrogels can be designed with patient-specific targeting capabilities based on tumor cell surface markers or patient genetic characteristics, allowing for precise drug delivery to tumor cells and improving therapeutic efficacy while reducing side effects (171). A pH-sensitive, tumor-specific targeted prodrug nanoparticle hydrogel featuring penetrating peptide modifications has demonstrated efficient suppression of tumor recurrence in postoperative breast cancer models, illustrating the potential of hydrogels for personalized postoperative cancer therapy (138).

Additionally, hydrogels can be utilized to develop personalized tumor vaccines. By utilizing patient-derived tumor antigens (such as neoantigens) and combining them with the sustained release properties and immune modulation functions of hydrogels, personalized tumor vaccines can be created to stimulate patients’ immune systems to combat tumors (172, 173). This hydrogel-based personalized cancer vaccine is expected to provide new strategies for postoperative cancer treatment, enhancing patient survival and quality of life. Moreover, the combination of hydrogels with nanotechnology can further enhance their functionalities in personalized cancer therapy, such as enabling multimodal treatment and real-time monitoring (174).

Beyond this, the convergence of hydrogel technology with AI and machine learning (ML) is opening new frontiers. AI algorithms can analyze high-throughput data from hydrogel libraries to predict optimal stiffness, degradation rate, and ligand density for a given patient’s TME (170, 175). When combined with microfluidic organoid-on-a-chip systems, AI-designed hydrogels can be rapidly validated using patient-derived tumor organoids, enabling truly personalized immunomodulatory therapy within a clinically relevant timeframe (176).

## Prospects of synergizing hydrogels with immunotherapy

Hydrogel-assisted immunotherapy provides a strategy to enhance local immune activation while limiting systemic exposure. Immunotherapy works by activating the patient’s own immune system to fight tumors, but it often faces challenges such as insufficient local immune responses and systemic toxic side effects. Hydrogels, as local delivery platforms, can improve the pharmacokinetics of immunotherapeutic agents, enhance local immune activation, and reduce systemic toxicity (177, 178). For example, an injectable hydrogel loaded with cyclic dinucleotides (CDNs) and molybdenum-based polyoxometalates (POMs) can achieve photothermal therapy while utilizing the ROS scavenging activity of POMs and the immune-stimulating effects of CDNs to mitigate inflammatory responses and enhance antitumor efficacy, with minimal side effects on normal tissues (179).

Nevertheless, hydrogel-assisted immunotherapy still faces several unresolved challenges. The improved therapeutic outcomes observed in experimental models may partly depend on tumor accessibility, implantation conditions, and experimental settings that may not fully reflect clinical scenarios. In addition, excessive immune activation may induce local inflammatory damage, whereas insufficient stimulation may fail to overcome immunosuppressive networks within the TME. Future studies should therefore define optimal combinations of hydrogel properties, immune modulators, and treatment schedules through standardized comparative approaches.

Beyond direct delivery of immunotherapeutic agents, hydrogels themselves or their loaded modulators can actively reshape the TME, such as alleviating immunosuppression and promoting vascular normalization, thereby improving the effectiveness of immunotherapy. For instance, hydrogels loaded with anti-angiogenic drugs and immune modulators can promote vascular normalization, enhance immune cell infiltration, and modulate the immunosuppressive TME, realizing a synergistic effect between anti-angiogenic and immunotherapeutic strategies (153). Moreover, the combined application of hydrogels with immune cells holds potential. For example, hydrogels loaded with effector immune cells (such as natural killer cells, or NK cells, and chimeric antigen receptor T cells, or CAR-T cells) can be precisely delivered to tumor sites, enhancing antitumor immune responses (180). These approaches may improve the local efficacy and safety of cancer immunotherapy.

An emerging combination is hydrogels loaded with oncolytic viruses (OVs). The hydrogel protects OVs from neutralizing antibodies, prolongs local viral replication, and reduces systemic toxicity (181). Another exciting direction is “closed-loop” smart hydrogels that sense TME biomarkers (e.g., lactate, pH, ROS) and autonomously adjust drug release or degradation behavior via embedded feedback circuits (e.g., enzyme-triggered or DNA strand displacement networks) (182, 183). Such systems could maintain an optimal anti-tumor inflammatory state without external intervention.

## Future directions of hydrogels in TME research

In the future, hydrogel research in TME will prioritize the creation of models that more accurately reflect these environments. By leveraging advanced materials science and bioengineering technologies, researchers can develop more complex and biomimetic hydrogel models. These models are expected to incorporate multiple cell types, gradient biochemical signals, and dynamic mechanical properties, enabling more precise replication of cell-cell and cell-matrix interactions, as well as various physical and chemical signals within the TME (111). This progression will provide more effective tools for comprehensive studies of tumor biology and the development of novel therapeutic strategies (184, 185).

Additionally, integrating hydrogels with emerging technologies will be a significant focus. For instance, combining hydrogels with AI and machine learning (ML) has the potential to enhance the analysis of large datasets, optimize hydrogel design and performance, and facilitate precise regulation of the TME for tailored treatments (186). Furthermore, metabolic reprogramming represents an important future direction for responsive hydrogel systems, as modulation of the metabolic state of the TME may suppress tumor growth and progression (187). Beyond targeting extracellular metabolites such as glucose and lactate, emerging biomaterial strategies suggest that intracellular metabolic and ionic homeostasis may also provide therapeutic opportunities. For example, a pH-responsive metal–phenolic nanocluster was reported to disrupt mitochondrial Ca²^+^ homeostasis and amplify ROS–Ca²^+^ crosstalk, thereby promoting mitochondrial dysfunction and tumor-cell apoptosis (188). Although this system is not itself a hydrogel, it provides a transferable design concept for incorporating stimulus-responsive intracellular metabolic regulation into future hydrogel platforms. Concurrently, the advancement of multifunctional intelligent hydrogel systems that integrate diagnostics, treatment, and real-time monitoring will provide more comprehensive solutions for cancer therapy (35, 189).

Another frontier is the use of hydrogels to modulate the gut–tumor axis. Oral or rectal administration of prebiotic hydrogels that reshape gut microbiota composition (e.g., promoting Akkermansia muciniphila) can reduce systemic inflammation and improve response to immune checkpoint inhibitors in colorectal and liver cancers (190). Hydrogels that selectively deliver short-chain fatty acids or bacterial metabolites to the tumor site are under early investigation (191, 192).

## Conclusions

Hydrogels offer a versatile and forward-looking platform for modulating inflammation within the TME. Advances in dynamic covalent chemistries, mechanotransduction targeting, and living materials have expanded their functionality beyond passive carriers to actively adaptive systems. However, long-term biosafety, degradation consistency, GMP-compatible manufacturing, and patient-specific heterogeneity remain formidable barriers. Looking ahead, next-generation hydrogel systems will likely evolve from passive delivery platforms toward intelligent, patient-adaptive biomaterials integrating AI, multi-omics analysis, and personalized biological models. AI-assisted biomaterial design may enable prediction and optimization of hydrogel parameters, including mechanical properties, degradation behavior, and ligand presentation, based on patient-specific tumor characteristics. Meanwhile, integration with patient-derived organoids, tumor-on-chip platforms, and multi-omics profiling may facilitate individualized hydrogel screening and precision immunomodulatory therapy. Furthermore, intelligent responsive hydrogels capable of sensing dynamic TME signals and adjusting therapeutic outputs through closed-loop regulation represent a promising direction for achieving adaptive control of tumor inflammation. Hydrogels are poised to become a core component of combination immunoregulatory strategies rather than a standalone tool.
